# Radiofrequency Ablation as an Effective Long-Term Treatment for Chronic Sacroiliac Joint Pain: A Systematic Review of Randomized Controlled Trials

**DOI:** 10.7759/cureus.26327

**Published:** 2022-06-25

**Authors:** Michael Lowe, Oluwasemilore Okunlola, Shafaat Raza, Stephen A Osasan, Sudiksha Sethia, Tayyaba Batool, Zarna Bambhroliya, Joel Sandrugu, Pousette Hamid

**Affiliations:** 1 Research, California Institute of Behavioral Neurosciences & Psychology, Fairfield, USA; 2 Neurology, California Institute of Behavioral Neurosciences & Psychology, Fairfield, USA

**Keywords:** sacroiliac joint, chronic pain, rhizotomy, denervation, neurotomy, radiofrequency ablation

## Abstract

Radiofrequency ablation (RFA) has emerged as a popular intervention for chronic pain management, including pain originating in the sacroiliac joint. It offers a less invasive option than surgery but with better results than the previous standard treatment with steroid and anesthetic injections. Procedure volumes have enjoyed significant growth in the market in recent years. The evidence supporting this intervention, in the form of randomized controlled trials, however, is both thin and mixed. The purpose of this systematic review is to evaluate the body of randomized controlled trials (RCTs) to determine the quality of support for and against the use of radiofrequency ablation to treat sacroiliac joint (SIJ) pain. Several important new papers have emerged since previous systematic reviews with similar objectives were published. The review was conducted according to PRISMA (Preferred Reporting Items for Systematic Reviews and Meta-Analyses) guidelines, and three databases were used: PubMed, Google Scholar, and Scopus. Only RCTs were sought, and no other filters, such as a historical timeline cut-off, were used. Among 95 publications that returned in response to the query, 16 were ultimately accepted as meeting the inclusion/exclusion criteria. The Cochrane risk-of-bias tool was utilized as a quality assessment measure, and the GRADE (Grading of Recommendations, Assessment, Development, and Evaluations) framework was used to assess the certainty of the evidence. Among the included publications, 15 out of 16 publications featured positive results and conclusions that supported the use of RFA in treating chronic sacroiliac joint pain. The single negative study was also the largest trial (n=681), but it was identified as “High Risk” using the Cochrane risk-of-bias tool. It included several design flaws including neither operator nor patient blinding, missing information, use of inconsistent treatment modalities across groups, and disproportionate drop-out rates. Despite its flaws, we have included this study in the present review because of its sheer size. Taken in aggregate, the total body of research included in this review supports this intervention. Questions continue to exist around whether there are clinically significant benefits associated with different RFA modalities (for example, unipolar vs. bipolar), with convincing evidence supporting each of them. Finally, it can be concluded that while the benefits are reasonably and justifiably supported in this patient population for up to one year, there is a dearth of evidence beyond a 12-month post-intervention follow-up.

## Introduction and background

The clinical use of radiofrequency ablation (RFA) devices is experiencing a historic high and continues to grow. With an estimated 11.5% Compounded Annual Growth Rate, the market is predicted to be worth $7.8 billion globally by 2027 [1]. Treatment for sacroiliac joint (SIJ) pain has been proposed over a continuum of care [2]. On the conservative end lie non-steroidal anti-inflammatory drugs (NSAIDs), opiates, physical therapy, durable medical equipment, and anesthetic and steroid injections. RFA offers the benefits of more aggressive approaches like surgery while maintaining the minimally invasive nature of the less intrusive treatments. 

**Figure 1:**
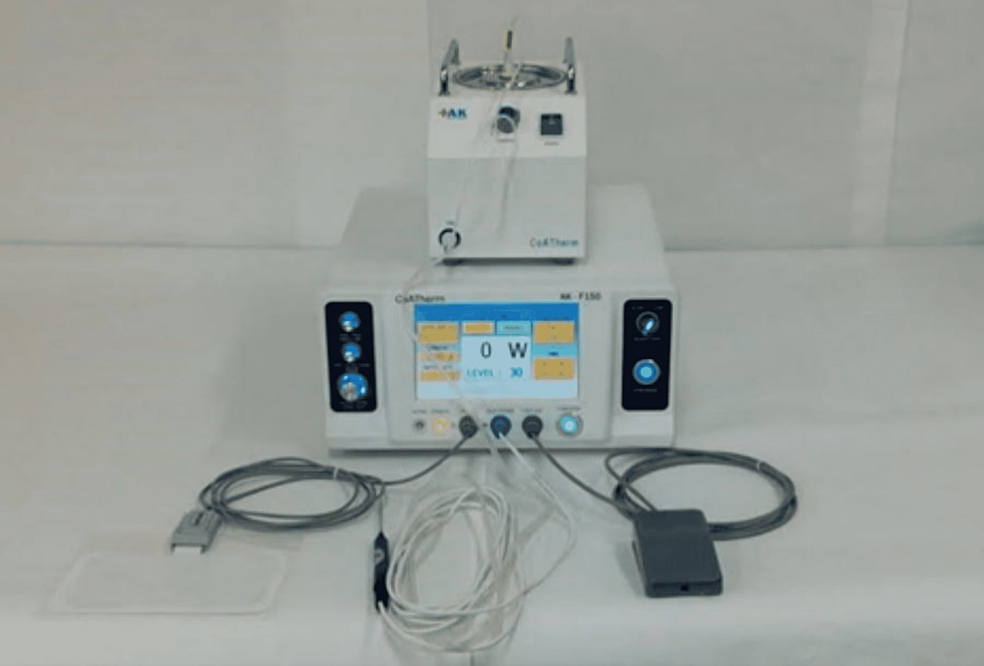
The radiofrequency ablation equipment The image of the ablation equipment is taken from [4] under the Creative Commons Attribution-Share Alike 4.0 International license.

RFA device systems typically consist of a generator, interface display panel, cannula, adapter, and grounding pad [3]. Figure 1 shows the equipment set up as it typically appears in the operatory. 

**Figure 2:**
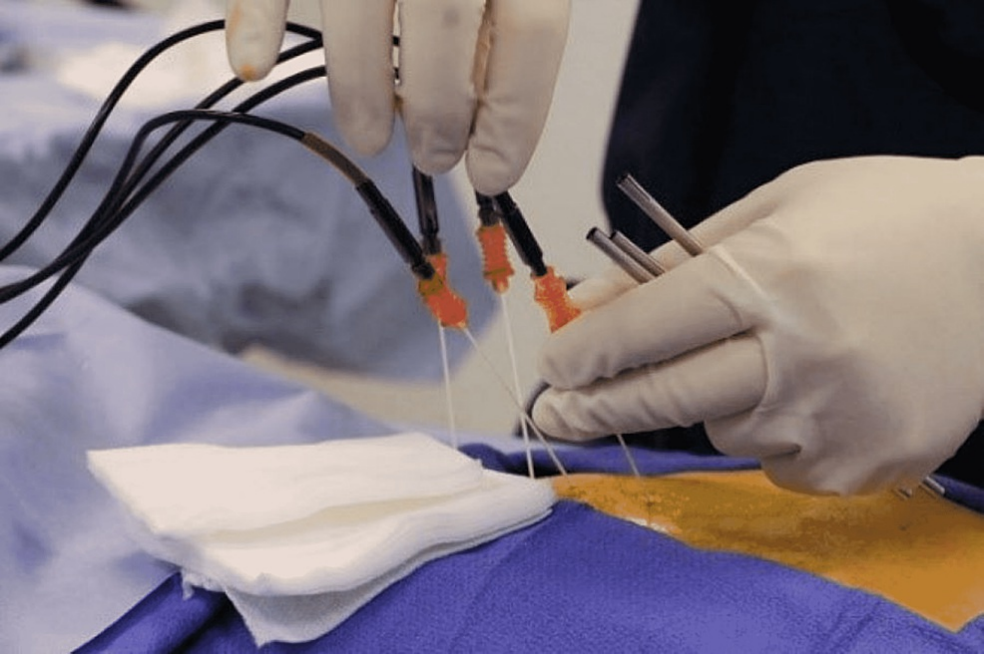
Procedural application of cannula The image of the radiofrequency ablation procedure is taken from [5] under the Creative Commons Attribution-Share Alike 4.0 International license.

 Figure 2 shows the procedural application.

Commonly reported traumatic causes of SIJ pain include pelvic ring fractures, soft tissue damage from falls, motor vehicle collisions, and heavy lifting that results in undue strain. Typical atraumatic triggers involve spondyloarthropathy, enthesopathy, infectious disease, osteoarthritis, scoliosis, and even pregnancy [6].

Shih et al. conducted a review of different RFA techniques for the management of both lumbar facet joint and SIJ pain [7]. While insightful, this review did not take into consideration the long-term (more than six months) clinical benefit that is being represented to patients in the market [8]. Sun et al., published a meta-analysis focused on cooled RFA for chronic SIJ pain [9]. Only seven studies were reviewed, and the key conclusions were centered around product safety. 

Della Volpe et al. argued that the efficacy data is “controversial” but that the rates of procedures performed continue to rise anyway [10]. They cited data indicating that the number of lumbar RFA procedures rose 131% between 2009-2016, a time period that did not feature much new research.

Our objective was to identify and evaluate randomized controlled trials (RCTs) published in peer-reviewed journals. Findings are of interest to healthcare professionals, patients and their families and caregivers, the insurance industry, medical device manufacturers, and compliance regulating authorities.

## Review

Methods

We searched for RCTs in which patients presenting with SIJ pain were treated with RFA. No historical timeline filters were used. Additionally, there were no filters used for study location, single-center vs. multi-center, setting, or patient population characteristics, including gender, age, race/ethnicity, or nationality. All articles screened were available in English only articles readily available in the full-text format were selected. Upon review, the intervention was verified as RFA (also referred to as neurotomy, rhizotomy, and denervation). There were no deviations from the pre-established strategy. The date of the last search was March 29, 2022.

PubMed

Keywords: radiofrequency ablation; ablation; radiofrequency neurotomy; neurotomy; radiofrequency denervation; denervation; radiofrequency rhizotomy; rhizotomy; chronic pain; pain; sacroiliac joint; sacrum; ilium.

Filters: “Randomized Controlled Trial” and “Clinical Trial” were selected as the publication type; all other fields in all other categories were deselected.

Google scholar

Keywords: radiofrequency ablation; sacroiliac joint; pain.

Filters: “Any time,” and, “Any type,” were selected; “include citations,” was selected; “include patents,” was deselected.

Scopus

Keywords: radiofrequency ablation; sacroiliac joint; pain.

Filters: the only filter selected was, “Type,” as, “Article Title, Abstract, Keywords, Authors.” All other filters were deselected.

The Cochrane risk-of-bias assessment tool was used [11]. Articles were included regardless of the final assessment because there is a lack of well-designed research on this topic. Additionally, the most common reasons for unfavorable assessment were patients lost to follow-up and concerns with the control. Similar numbers were lost from each of the treatment and control groups. As for the two trials that had issues with the control design, both were relatively small.

Results

**Figure 3:**
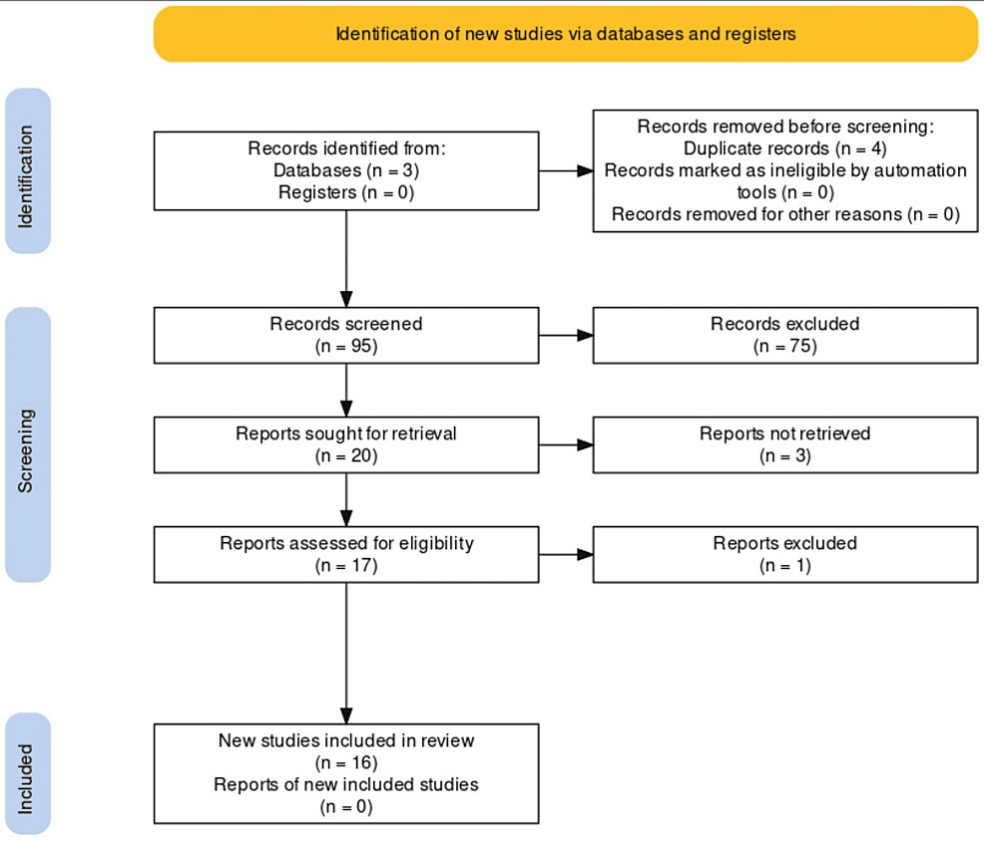
Flow diagram showing the number of citations that were returned upon the initial query, and the number of final articles included in the review

Ninety-nine citations were returned: Scopus (83), PubMed (9), and Google Scholar (7). Twenty articles were retrieved after screening for duplicates and verification of publication type: Scopus (13), PubMed (7), and Google Scholar (0). Three articles were excluded due to lack of full-text format, and an additional article was excluded for deviation from the publication type to which it was categorized, resulting in 16 final articles for review (Figure 3). 

**Figure 4:**
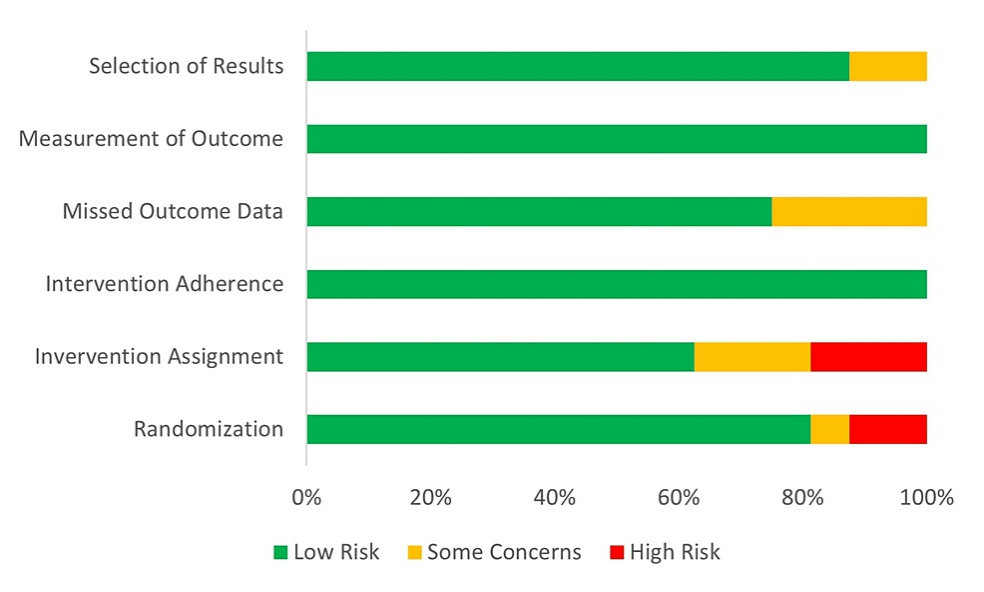
Risk of bias

The risk of bias summary is included in Figure 4. 

The details of the included studies details are summarized in Table 1, including a description of the measurement scales and tools used.

**Table 1:** Details of the included studies and measurement scales used in them

Trial	Country	Numerical Rating Scale (NRS)	Oswestry Disability Index (ODI)	Global Pain Evaluation (GPE)	Visual Analog Scale (VAS)	Neck Disability Index (NDI)	Roland Morris Disability Questionnaire (RMD)	Patient Global Impression of Change (PGIC)	Short Form-36 Bodily Pain (SF36-BP)	Short Form 36 Physical Functioning (SF36-PF)	Other
Juch et al. [12]	The Netherlands	*	*	*							Health-related Quality of Life (EuroQoI 5D Health Questionnaire), general health (RAND 36-item Health Survey), and chronic pain experiences (West Haven-Yale Multidimensional Pain Inventory)
Dutta et al. [13]	India	*	*	*							
Shustorovich et al. [14]	United States		*			*					
Cohen et al. (2022) [15]	United States	*									
Salman et al. [16]	Egypt				*						Reduction in analgesic consumption
Abo Elfadl et al. [17]	Egypt	*	*								
Zheng et al. [18]	China				*						
Cohen et al. (2008) [19]	United States	*	*	*							
Patel et al. (2012) [20]	United States		*						*	*	Quality of Life Assessment, Treatment Success
Patel (2015) [21]	United States	*	*						*	*	
Nath et al. [22]	Sweden				*						Global Perception of Improvement, Quality of Life (both patient-reported on scale of 1/LEAST to 6/MOST); Range of Motion Lumbar Spine/Hip Movement (in degrees with goniometer); Clinical Signs (measured as +/-)
Chou et al. [23]	Taiwan		*		*			*			
Mehta et al. [24]	United Kingdom	*									
Bayerl et al. [25]	Germany	*	*				*		*		Odom's Criteria
Burnham et al. [26]	Canada										Custom questionnaire evaluating pain intensity and frequency, analgesic intake, disability, satisfaction (with current pain level and the procedure), and procedure complications

Table 2 summarizes the common pain measurement scales and tools used in the RCTs evaluated in this review.

**Table 2:** Summary of measurement scales and tools used for chronic pain Abbreviations: Numerical Rating Scale (NRS), Oswestry Disability Index (ODI), Global Pain Evaluation (GPE), Visual Analog Scale (VAS), Neck Disability Index (NDI), Roland Morris Disability Questionnaire (RMD), Patient Global Impression of Change (PGIC), Short Form-36 Bodily Pain (SF36-BP), Short Form 36 Physical Functioning (SF36-PF).

Scale	Full Name	Description
NRS	Numerical Rating Scale	Patients are asked to rate average pain intensity over last 7 days by selecting a single number from 0 to 10
ODI	Oswestry Disability Index	4 versions exist; questionnaire grouped into topic sections, with examples being pain intensity, personal care, and lifting
GPE	Global Perceived Effect	11-point format that represents a compromise between discriminative capacity, reliability, and patient preference
VAS	Visual Analog Scale	Patients are asked to make a hatch mark on a 100-mm line that represents average pain intensity over the last 7 days
NDI	Neck Disability Index	Self-reported questionnaire focused on psychometric properties, designed to detect very small increments of change
RMD	Roland Morris Disability Questionnaire	Questionnaire focused on daily physical activities/functions such as housework, sleeping, mobility, dressing, getting help, appetite, irritability, and pain severity
PGIC	Patient Global Impression of Change	7-point scale of ‘very much worse’ to ‘very much improved' and interpreted as disease deterioration, stable disease, or disease improvement vs. baseline
SF36-BP	Short Form 36 - Bodily Pain	Questionnaire instrument developed for use with primary care and chronic disease patients
SF36-PF	Short Form 36 - Physical Functioning	Broad-based, generic evaluation measure that considers several domains of physical functioning

Only a single study stated follow-up assessment at the unconventional post-treatment intervals of one week (Chou et al.), two weeks (Dutta et al.), three weeks (Juch et al.), six weeks (Juch et al.), and two months (Shustorovich et al.), as seen in Table 3. More popular intervals were, three and six months (12 studies each), followed by one month (10 studies). The most noteworthy finding is the glaring absence of any studies tracking patients beyond 12 months. Finally, Juch et al. represented the most judicious follow-up of any individual publication reviewed, with a total of six post-treatment assessments spanning from 21 days to 12 months.

**Table 3:** Follow-up timeframes

Trial	7 d	15 d	21 d	1 M	1.5 M	2 M	3 M	6 M	9 M	12 M
											Dutta et al. [13]		*		*			*	*		
Zheng et al. [18]							*	*		
Shustorovich et al. [14]				*		*				
Cohen et al. (2008) [19]				*			*	*		
Patel (2015) [21]										*
Patel et al. (2012) [20]				*			*	*	*	
Nath et al. [22]								*		
Cohen et al. (2022) [15]				*			*			
Terao et al. [27]										*
Bayerl et al. [25]				*			*	*		*
Mehta et al. [24]							*	*		
Salman et al. [16]				*			*	*		
Burnham et al. [26]				*			*	*	*	*
Juch et al. [12]			*		*		*	*	*	*
Abo Elfadl et al. [17]				*			*	*	*	*
Chou et al. [23]	*			*			*	*		

Five of sixteen articles claimed no external funding. Three articles did not disclose any information. Of the remaining eight, the two Patel trials were funded by a manufacturer, leaving six funded by grants. Juch et al. was also funded by unspecified, “Dutch insurance companies.” It is interesting that the only trial funded by an insurance company was the only one that concluded no clinical benefit.

Table 4 summarizes the control arm(s) across the publications reviewed. Placebo/sham RFA and steroid injections were most popular, with five trials each. 

**Table 4:** Summary of the control arm interventions

Trial	Control/Comparison
		Dutta et al. [13]	Intraarticular methylprednisolone
Zheng et al. [18]	Celecoxib treatment (400 mg/day)
Shustorovich et al. [14]	Dexamethasone 4 mg/mL
Cohen et al. (2008) [19]	Placebo/sham radiofrequency injection
Patel (2015) [21]	Placebo/sham radiofrequency injection
Patel et al. (2012) [20]	Placebo/sham radiofrequency injection
Nath et al. [22]	Placebo/sham radiofrequency injection
Cohen et al. (2022) [15]	(2) Steroid injections (10 mg dexamethasone), medial branch block (0.5 mL 0.5% bupivacaine)
Terao et al. [27]	RFA treatment plus any combination of: piriform muscle block, botulinum toxin injection, spinal cord stimulation
Bayerl et al. [25]	Monopolar vs. bipolar radiofrequency ablation (RFA)
Mehta et al. [24]	Placebo/sham radiofrequency injection
Salman et al. [16]	Steroid injection (40 mg/ml depot methylprednisolone)
Burnham et al. [26]	No control
Juch et al. [12]	Standardized exercise program
Abo Elfadl et al. [17]	Intraarticular methylprednisolone alone, 30 mg (treatment was same steroid injection + RFA)
Chou et al. [23]	Cooled RFA in both arms, but each with a different diagnostic procedure

We conducted a difference of means and standard deviation analysis to investigate RFA vs. sham control. Studies were isolated with the greatest number of shared parameters for the type of control, measurement tool used, and follow-up interval. Five studies investigated both the Oswestry Disability Index (ODI) and Numeric Rating Scale (NRS), over follow-up intervals of one, three, and six months: Dutta et al., Cohen et al. (2008), Patel et al., Abo Elfadl et al., and Bayerl et al. Bayerl et al. was removed as there was no sham control (mono-lesion vs. multi-lesion).

Data were missing or not reported for three of the six measurement index tool/follow-up time interval combinations: ODI at one month (Dutta et al.), ODI at six months (Cohen et al. (2008) and Patel et al.), and NRS at six months (also Cohen et al. (2008) and Patel et al.). These findings are summarized in Table 5.

**Table 5:** ODI and NRS mean scores and standard deviations at one, three, and six months of follow-up for all publications reporting such data

		Oswestry Disability Index (ODI)						Numerical Rating Scale (NRS)					
		1 M	±	3M	±	6 M	±	1 M	±	3M	±	6 M	±
														Dutta et al. [13]	Treatment	-	-	9.1	3.5	8.0	3.7	2.9	0.6	3.1	0.9	3.2	1.2
	Control	-	-	12.1	4.5	13.1	4.3	3.3	0.5	4.4	1.0	5.4	1.5
Cohen et al. (2008) [19]	Treatment	20.9	10.9	18.5	11.6	22.6	10.6	2.4	2	2.4	1.5	2.6	2.2
	Control	43.6	14	24	8.5	-	-	6.3	2.4	6	0	-	-
Patel et al. (2012) [20]	Treatment	25	14	26	17	24	16	3.4	2.6	3.7	2.7	3.6	2.6
	Control	31	11	37	6	-	-	4.1	2	5	2.4	-	-
Abo Elfadl et al. [17]	Treatment	22	13.75	21.5	11.5	20	11.25	3	1	2	1.5	1.5	1.25
	Control	40	11.75	34.5	12.5	27.5	12.5	2.5	2.5	3.5	2.5	3.5	2.5

Calculations were performed for the three remaining measurement indices and follow-up time interval combinations: ODI at three months and NRS at one and three months. The results are in Table 6.

**Table 6:** Differences between means and the associated standard deviations, for ODI at the one month and NRS at one and three months of follow-up, for all publications reporting such data

	DIFFERENCE OF THE MEANS					
	Measurement Scale and Time Interval					
	Oswestry Disability Index (ODI) 3-month		Numerical Rating Scale (NRS) 1-month		NRS 3-month	
Dutta et al. [13]	3.0	± 2.3	0.4	± 1.5	1.3	± 2.1
Cohen et al. (2008) [19]	5.5	± 13.4	3.9	± 3.5	3.6	± 4.3
Patel et al. (2012) [20]	11	± 5.4	0.7	± 1.6	1.3	± 2.1
Abo Elfadl et al. [17]	13	± 7.3	-0.5	± 0.8	1.5	± 0.8

**Figure 5:**
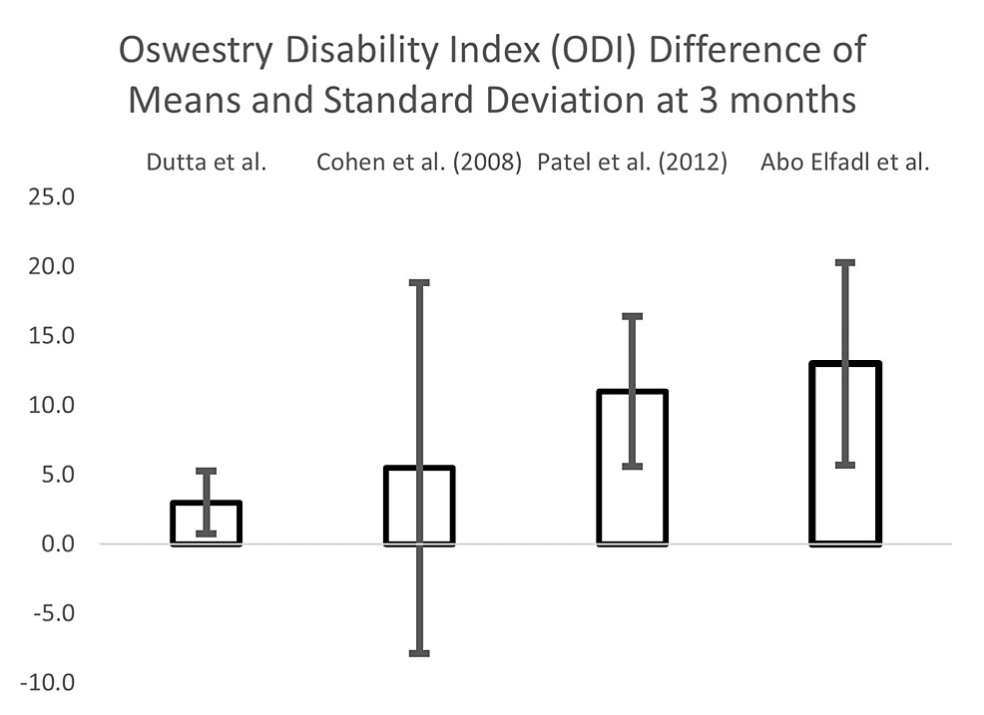
Difference of means and associated standard deviations at three months according to the Oswestry disability index (ODI) Dutta et al. [13], Cohen et al. (2008) [19], Patel et al. [20], Abo Elfadl et al. [17]

The ODI difference of means and the associated standard deviations at three months can be seen in Figure 5. 

**Figure 6:**
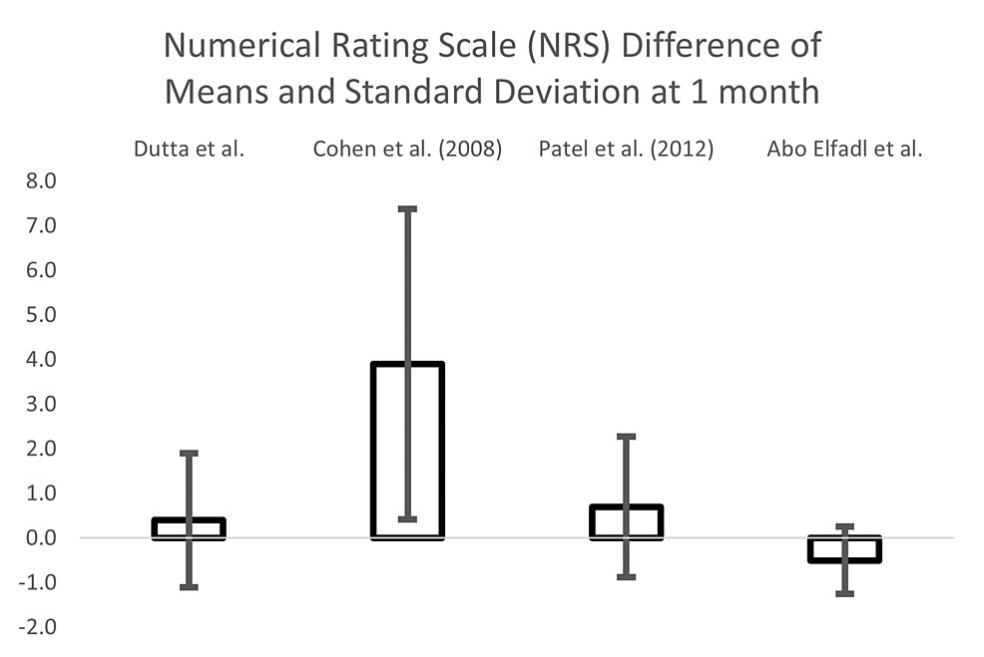
The numerical rating scale (NRS) difference of means and associated standard deviations at one month Dutta et al. [13], Cohen et al. (2008) [19], Patel et al. [20], Abo Elfadl et al. [17]

The NRS difference of means and the associated standard deviations at one month can be seen in Figure 6. 

**Figure 7:**
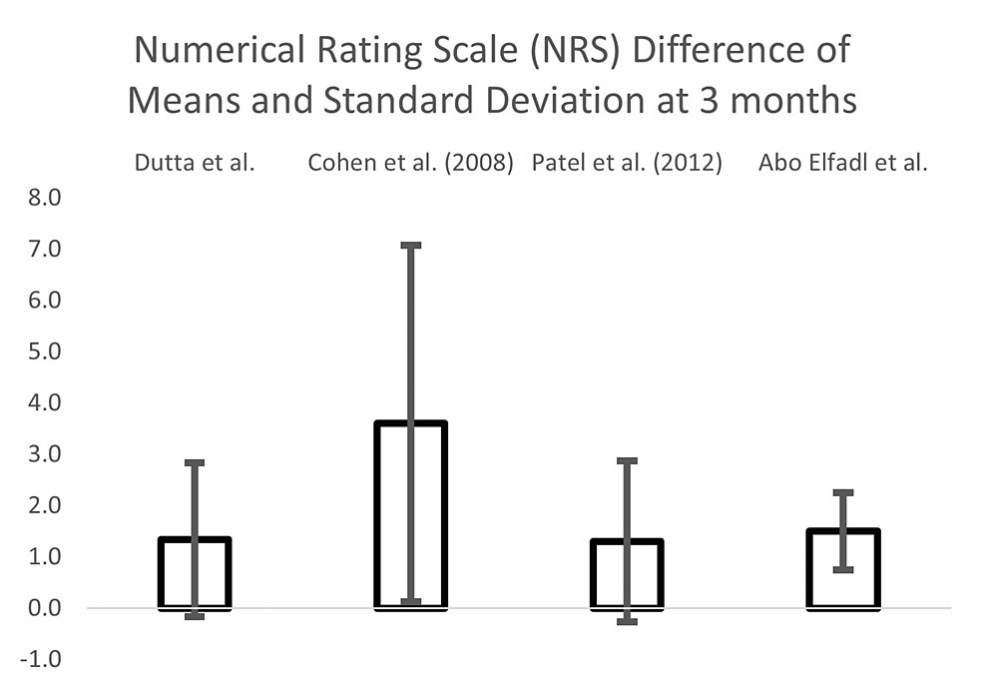
The numerical rating scale (NRS) difference of means and associated standard deviations at three months Dutta et al. [13], Cohen et al. (2008) [19], Patel et al. [20], Abo Elfadl et al. [17]

The NRS difference of means and the associated standard deviations at three months can be seen in Figure 7. 

Assessment of the certainty of the evidence

The GRADE approach is widely accepted as a methodology for assessing certainty [28]. Our review featured a net certainty assessment score of 3, reported as “+++ Moderate," in accordance with the GRADE guidelines (Table 7).

**Table 7:** Assessment of certainty according to the GRADE framework (Grading of Recommendations, Assessment, Development and Evaluations)

	GRADE Parameter	Score	Justification for the assigned score
Domains	Risk of bias	0	Only RCTs were reviewed
	Inconsistency	0	There was variability in the subjective measurement tools used
	Indirectness	-1	PICO elements (patients, intervention, comparison, or outcome) did not exactly match the articles assessed in this review
	Imprecision	0	A variety of timeframes and measurement tools were used
	Publication bias	0	The a priori search strategy did not influence the results; very few filters were used
Factors	Dose-response gradient	0	Supra-therapeutic levels do not apply to the RFA intervention
	Large size effect	2	Only RCTs were used in this review
	Plausible residual confounding	2	No evidence that confounding factors existed

Discussion

Several themes emerged in our review. First, RFA is typically compared to some other traditional treatment approach. It is challenging and unethical to limit a control group of patients in pain to no treatment. Second, studies used combinations of one, three, six, and 12 months of follow-up. Finally, methodologies for measuring pain are subjective. While each of the different scales has its advantages and disadvantages, and no single measurement tool has emerged as superior in all situations, they are well-accepted and routinely used [29]. Of the 16 clinical trials evaluated, 15 concluded that there was a clinical benefit. Conclusions can be referenced in Table 8. 

**Table 8:** Conclusions and summaries of the evaluated clinical trials Abbreviations: Numerical Rating Scale (NRS), Oswestry Disability Index (ODI), Global Pain Evaluation (GPE), Visual Analog Scale (VAS), Neck Disability Index (NDI), Roland Morris Disability Questionnaire (RMD), Patient Global Impression of Change (PGIC), Short Form-36 Bodily Pain (SF36-BP), Short Form 36 Physical Functioning (SF36-PF).

Trial	Year	Journal	n =	Primary Endpoint	Conclusion
Dutta et al. [13]	2018	Pain Physician	30	Numerical Rating Scale (NRS) (0–10) pain score, which was evaluated both prior to receiving the treatment and post-procedure at 1-, 3-, and 6-month intervals.	This comparative study shows that pulsed radiofrequency denervation of the L4 and L5 (lumbar) primary dorsal rami and S1 - S3 (sacral) lateral branches provide significant pain relief and functional improvement in patients with sacroiliac joint pain.
Zheng et al. [18]	2013	Rheumatol Int	155	Global pain intensity in visual analog scale (VAS) at week 12.	This trial showed that palisade sacroiliac joint radiofrequency neurotomy is superior to celecoxib in reducing global pain intensity, and improving functional and mobility, with minimal concern for safety issue if carried out properly.
Shustorovich et al. [14]	2021	Pain Physician	63	4- and 8-weeks post-intervention to evaluate the incidence of post-procedure pain (questionnaire) and function using the Oswestry Disability Index (ODI) or the Neck Disability Index (NDI).	A statistically significant reduction in post-neurotomy pain was observed in the steroid group.
Cohen et al. (2008) [19]	2008	Anesthesiology	28	0 – 10 NRS pain score, which reflected the average pain experienced by the patient for 10 days before follow-up.	Preliminary evidence that L4 and L5 primary dorsal rami and S1 – S3 lateral branch radiofrequency denervation may provide intermediate-term pain relief and functional benefit in selected patients with suspected sacroiliac joint pain.
Patel [21]	2015	Pain Practice	51	Long-term outcomes (NRS, ODI, Short Form-36 Bodily Pain [SF-36BP]) of cooled radiofrequency (CRF) lateral branch neurotomy (LBN) as a treatment for sacroiliac (SI) region pain.	These favorable 12-month results illustrate the durability of effective CRF/LBN-mediated treatment of SI region pain for selected patients.
Patel et al. [20]	2012	Pain Medicine	51	Pain (numerical rating scale, SF-36BP), physical function (Short Form-36 Physical Functioning [SF-36PF]), disability (ODI), quality of life (assessment of quality of life), and treatment success.	The treatment group showed significant improvements in pain, disability, physical function, and quality of life as compared with the sham group (duration and magnitude of relief extending beyond 9 months).
Nath et al. [22]	2008	Spine	40	Global perception of improvement, relief of generalized pain, low back pain, and pain in the lower limb.	Radiofrequency facet denervation is not a placebo and could be used in the treatment of carefully selected patients with chronic low back pain.
Cohen et al. (2022) [15]	2022	Reg Anesth Pain Med	346	Change in patient-reported average pain intensity on a numerical rating scale (average NRS) using linear regression at 1 and 3 months.	Identifying treatment responders is a critical endeavor for the viability of procedures in LBP. Patients with greater disease burden, depression and obesity are more likely to fail interventions.
Terao et al. [27]	2020	Neurological Science	16	Duration required for improvements in lower back pain by more than 50% (numerical rating scale ≤ 5).	Multimodal treatment including facet joint denervation is safe and relatively effective in patients with neuromuscular disorder (NMD)-associated kyphoscoliosis.
Bayerl et al. [25]	2018	Neurosurgical Review	64	1, 3, 6 and 12 months after RFA; numeric pain rating scale (NPRS), Roland Morris Disability Questionnaire, ODI, and Odom’s criteria, Short Form 36 score.	An improvement of operating time, x-ray time as well as of the clinical outcome 1 year after RFD in patients treated with the multiple lesion probe (a clear advantage compared to a conventional monolesion RFA of the SIJ).
Mehta et al. [24]	2018	Pain Physician	30	Mean NRS-11 score at 3 months post-treatment.	Radiofrequency neurotomy using a strip lesioning device is an appropriate therapy to treat SIJ pain.
Salman et al. [16]	2016	Egyptian Journal of Anaesthesia	30	> 50% pain relief at 1-, 3-, and 6-months post-intervention.	Radiofrequency ablation at L4 and L5 primary dorsal rami and S1 – S3 lateral sacral branch may provide effective and longer pain relief compared to the classic intra-articular steroid injection, in properly selected patients with suspected sacroiliac joint pain.
Burnham et al. [26]	2007	Regional Anesth. & Pain Medicine	7	Pain intensity and frequency, analgesic intake, disability, satisfaction, and procedure complications questionnaire at 1, 3, 6, 9, and 12 months post-treatment.	RF sensory ablation of the SIJ using bipolar strip lesions is a technically uncomplicated and low-risk procedure. The resulting effects on pain, disability, and satisfaction are promising.
Juch et al. [12]	2017	JAMA	681	Pain intensity (numeric rating scale, 0-10; whereby 0 indicated no pain and 10 indicated worst pain imaginable) measured 3 months after the intervention.	In 3 randomized clinical trials of participants with chronic low back pain originating in the facet joints, sacroiliac joints, or a combination of facet joints, sacroiliac joints, or intervertebral disks, radiofrequency denervation combined with a standardized exercise program resulted in either no improvement or no clinically important improvement in chronic low back pain compared with a standardized exercise program alone.
Abo Elfadl et al. [17]	2022	Egyptian Journal of Anaesthesia	60	NRS, ODI, and Patient Global Impression of Change Scale (PGIC) before the intervention, and post-intervention at 1-, 3-, 6-, 9-, and 12-months.	RFA with methylprednisolone injection is a safe and efficient treatment for sacroiliac pain.
Chou et al. [23]	2022	Diagnostics	41	Improvements in VAS or ODI score at 1-week to 6-month follow-up visits.	This new strategy (cooled radiofrequency ablation) could be successfully adopted for rapid diagnosis of the source of comprehensive lower back pain.

There was one unexpected finding in the literature. The single article that refuted the beneficial outcome and concluded no improvement was also the largest (n=618), arguably published in the most reputable journal (Journal of the American Medical Association, JAMA), and perhaps the most robust (data was aggregated from three separate trials). Juch et al. found that “… radiofrequency denervation … resulted in either no improvement or no clinically important improvement in chronic low back pain … [12].” The stated limitations were significant; for example, neither patients nor investigators were blinded.

Radiofrequency ablation and steroid injections: competitors or partners?

There has been precedent for the use of steroid injections as a treatment for sacroiliitis [30-32]. Dutta et al. investigated RFA as an intervention compared to intraarticular methylprednisolone. Results were in favor of RFA and specifically saw NRS scores holding steady in this group, as opposed to unfavorably rising in the steroid injection group, at both three and six months. ODI scores were also favorable for the RFA group [13].

Shustorovich et al. took a different approach and used the two treatments together: RFA patients were administered steroid injections. The ODI was improved (p < 0.001) in the steroid sample (vs. a saline sample in the same patients, allowing them to serve as their own controls). It can therefore be concluded that these modalities together are a successful treatment [14]. 

Cohen et al. compared outcomes for expanded treatments for back pain: epidural steroid injections (for sciatica), SIJ injections, and facet interventions such as RFA. Included were 346 patients at seven hospitals [19]. Contrary to Dutta et al., this investigation resulted in no clear winner. All groups reported a decrease in average NRS (p<0.0001), however, there were no differences in change in average NRS across procedural groups (p=0.50). The most compelling finding of this article was that identifying responders based on patient profile attributes and medical history characteristics is of critical importance. This dimension has not been explored in other research to our knowledge. Specifically, the treatment was more likely to fail in patients presenting with obesity, depression, and other disease comorbidities[15].

Salman et al. conducted a comparison between RFA and steroid blocks to treat SIJ pain [16]. It assessed 30 patients, randomized to either RFA of L4, L5 (lumbar) primary dorsal rami and S1, S3 lateral sacral branch, or steroid injection under fluoroscopy. At the three post-intervention measurement intervals (one, three, and six months), 73%, 60%, and 53% of patients, respectively, reported ≥50% pain relief with RFA, vs. only 20%, 0%, and 0%, respectively, in the steroid injection group. These authors also touch on Cohen’s “personalized/responder” theme in the conclusion, describing positive outcomes in, “properly selected patients,” but there is no elaboration.

Salman et al., contribute an additional point, noting that the duration of pain relief is most likely tied directly to the physiological limits surrounding nerve tissue regeneration [16]. This duration has previously been posited as taking between six and twelve months [33]. This finding opens the door for innovation in the area of biological medicine, for example, enhancing the beneficial effects of RFA through selectively slowing the regrowth of the ablated nerve.

Rather than compare the two interventions in isolation, Abo Elfadl et al. looked at the combination of intra-articular pulsed radiofrequency with methylprednisolone injection vs. steroid injections only [17]. NRS and ODI were used, with follow-up time frames of 1, three, six, and 12 months. It was larger than others with n=60. The use of RFA resulted in a favorable reduction of NRS (vs. steroid injections alone) at all timeframes beyond one month. The conclusion describes, “[an] improve[ed] physical and mental quality of life,” although there were no empirical measures tied to the study to support this assertion.

Zheng et al., compared sacroiliac joint RFA to treatment using celecoxib, a cyclooxygenase-2 selective non-steroidal anti-inflammatory drug (NSAID) [18]. The use of NSAIDS to treat mild to moderate pain is well documented and commonly deployed [34]. This study included 155 patients, randomly assigned to receive either RFA or celecoxib (400 mg/day for 24 weeks), with effectiveness measured at 12 weeks using a VAS. RFA pain reduction was superior at both 12 and 24 weeks, and RFA was also more effective in improving physical function and spinal mobility. 

There are several RCTs to support the thesis that RFA is superior to steroid injections for the treatment of SIJ-associated lower back pain, and that the two interventions used together are also effective.

Supporting evidence from sham control trials

There is also evidence in the form of placebo-controlled studies. One of the earliest publications (2008) was also authored by Cohen. Success was measured as 50% or greater pain relief at one, three, and six months. Results were favorable with an exemplary finding of 80% of RFA patients vs. only 14% of placebo patients above the improvement threshold at one month [19].

Patel et al. published a study reporting on 51 patients with chronic SIJ pain [20], and later a 12-month follow-up on the same cohort [21]. Patients were randomized to either RFA or sham, with follow-up at one, three, and six months. Sham subjects were allowed to crossover at three months. Outcomes were measured using Short Form-36 Bodily Pain (SF-36BP), Short Form-36 Physical Functioning (SF-36PF), and ODI. RFA patients showed statistically significant improvements in pain, disability, and physical function with one result being 57% success (RFA) vs 12% success (sham) at three months.

In the follow-up study, the assessment was extended to 12 months, and the results maintained favorability for RFA. In the original RFA treatment group, a 2.7-point drop in the NRS score, a 13.9 decrease in the ODI, and a 15.8 increase in SF-36BP were observed - all favorable. In the crossover cohort (patients who began in the placebo group but accepted an opportunity at three months to switch to RFA), six-month outcomes were also favorable. A difference related to this trial and its follow-up is the specific use of cooled RFA. It has been proposed that cooling allows greater energy deposition into the nerve tissue and anatomical region, resulting in a larger effective lesion radius [35]. 

Nath et al., contributed findings on 40 patients, randomly assigned to RFA or sham [22]. The authors described a zygapophyseal joint procedure, which is anatomically distinct from the sacroiliac joint, but frequently reported with a similar description in terms of the pain originating from each area. The RFA patients showed improvement not only in back and leg pain but also in back and hip movement. 

Innovation

A variety of derivative RFA procedures have also been described. Chou et al. reported on cooled RFA with a rapid diagnosis protocol [23]. This trial demonstrates successful RFA outcomes that are enhanced when a more specific diagnosis can be confirmed. Two methods were used to assess SIJ pain patients (facet joint pain patients were also included) for more than three months: Technetium Tc99m methylene diphosphonate single-photon emission tomography/computed tomography and a modified Fortin finger test. Outcomes were measured using the visual analog scale (VAS) and ODI, at one week, and one, three, and six months. Over 70% of the 41 patients had greater than or equal to 50% reduction in VAS and ODI scores. It was the only study to report on the one-week post-intervention timeframe. The importance is that patients draw conclusions on the effectiveness of treatment early, which may impact compliance [36].

Mehta et al., published on strip lesioning [24]. It is described as the placement of a single electrode, as well as a three-point design, that results in the formation of five overlapping lesions. The logic is that larger lesion-area access can lead to improved, more efficient results. At three months, the mean RFA NRS-11 score had decreased from 8.1 to 3.4 (P < 0.001). The sham group saw no NRS-11 score benefit. This study was the only one that looked at the correlation with anxiety. The RFA group moved from baseline anxiety (9.4 ± 5.9) on the designated scale to no anxiety (6.6 ± 6.3) at three months, and the results were similar at six months. A limitation was that only 17 of the 30 enrolled patients participated. Strip lesioning has the potential to lead to improved industrial product design. Mehta separately co-authored a 12-month retrospective follow-up study on this approach, which reported an improvement in pain scores [37]. 

Bayerl et al, compared classical monopolar RFA to a device with a multi-electrode design. It has been asserted that unipolar RFA is prone to a high recurrence rate. A total of 121 patients were randomized to either the monopolar device design group (57) or the multipolar group (64) [25]. Follow-up intervals were one, three, six, and 12 months, and included Numerical Pain Rating (NPR), ODI, and Short Form-36 (SF-36). Results at a threshold of > 50% NPR pain reduction indicated a clinically successful outcome. This level was achieved by 72% of the multipolar users vs. only 39% of the traditional unipolar users. While the superiority of the multipolar design is striking, one is left to wonder why only 39% of the traditional users reported success. This finding, in isolation, is inconsistent with what has been reported by others [16,17,22,35].

Burnham et al., published a single cohort (n=37) pilot study on longitudinal axis SIJ RFA offering promising results. A successful outcome was defined as 50% or more survey-based pain reduction at three and six months. It was unique in the comparison of the longitudinal axis SIJ RFA procedure vs. the traditional (palisade) RFA technique. The only conclusion was that longitudinal axis RFA required greater procedure time but less fluoroscopy time vs. the palisade technique [26]. The practical benefit of less fluoroscopy time was not quantified and is not clear for either operator or patient. The greater procedure time may even be a disadvantage

The big outlier

In 2017, Juch et al. concluded that RFA is not an effective treatment for low back pain patients [12]. This article was contrary to all previous research literature.

Their series of three studies added RFA to a standardized exercise program (an aspect not seen in previous publications). They were enrolled at 16 sites in The Netherlands. Out of the 681 participants, 238 qualified based on a diagnostic SIJ block. The outcome measurement was less sophisticated: pain on a “0 - 10” scale at three and 12 months, and the benefit was designated as a positive change of two or more points. The conclusion of no benefit (vs. exercise alone), as well as a statement that “the findings do not support the use of radiofrequency denervation,” was based on mean differences of less than 2 at three months, with the SIJ cohort reporting a difference of -0.99.

The most significant limitation stated by the authors was that neither participants nor treating doctors were blinded. Different treatment approaches were used - such as multi-lesioning and cooled RFA - and no subgroup analysis was performed. The differences in these intervention approaches are considered so significant that, in fact, it is the very basis for the inclusion of other articles in our review [18,20,21,26]. Bayerl even noted a two-times difference in the benefit between two of the different approaches used in the Juch trials (traditional vs. multi-lesion) [35]. While the magnitude and detail exhibited by the Juch article are impressive, the increased level of bias must be interpreted with great care. 

Special populations

Two of the papers specified specific patient profiles. Zheng et al. focused on patients with ankylosing spondylitis in comparing RFA vs. NSAIDs [18]. Terao et al. investigated SIJ pain in patients with kyphoscoliosis [27]. It is worth emphasizing the importance of identifying “responders” that were brought forth by Cohen [15], as pre-existing conditions and medical history likely play a critical role in the outcome of any intervention. Terao investigated 22 anatomical sites from 16 patients, randomized into treatment (RFA + facet joint denervation) or control (facet joint denervation alone). The patients were followed for 48 weeks. The outcome was interesting and represented a deviation from other articles identified by this review: the duration of improvement of > 50% (5-point rating scale) was measured. This effective period was more favorable for the RFA group (30.7 weeks, vs. only 8.4 weeks, a duration improvement of nearly 4x). The benefit of 30.7 weeks of pain relief lands between six and 12 months and is consistent with other findings relating to RFA use and benefit [13,14,17,29,30,33].

Limitations

The decision was made to exclude any form of publication outside of RCTs. Other publication forms such as case reports, case series, retrospective studies, and observational studies were not included and may have been valuable. 

Lower back pain is a complicated clinical presentation. While the aim was to focus on SIJ pain publications (reflected in the keyword strategy), it is possible that research literature exists which investigates this patient profile, but without specifically referencing it as such. Put in analogous diagnostic lingo, this review features high specificity, but perhaps low sensitivity.

None of the studies included post-intervention assessments beyond 12 months. 

## Conclusions

The evidence evaluated in this review supports RFA as an intervention for chronic SIJ pain for periods of up to one-year post-treatment. Of the 16 RCTs, 15 showed positive results. The single trial that showed no difference between the treatment group and the control groups happened to be the largest trial. But it exhibited a high risk of bias. Unfortunately, what continues to be absent from the body of literature is a definitive, large-scale RCT demonstrating positive outcomes.

There is currently no consensus around the superiority of strip-lesioning vs. monopolar RFA. Convincing studies support each of them. The use of these additional features of RFA systems, therefore, must be left to operator preference and situational analysis of perceived benefits vs. increased costs.

The industry and market care little about the level of evidence. While not uncommon, it would seem that practitioners are making decisions based on experience and anecdotes. The number of procedures performed is far outpacing levels of research. 

It must be emphasized that the efficacy of this procedure-particularly over the long term-is likely to be influenced by factors such as lifestyle, medical history, and comorbidities among the candidate patients.
